# Supplementary material for: Characterization of In Vitro 3D Cell Model Developed from Human Hepatocellular Carcinoma (HepG2) Cell Line
Source: Cells. 2020 Nov 28;9(12):2557. doi: 10.3390/cells9122557 (PMC7759933; doi:10.3390/cells9122557)
Supplement: Supplementary file 1 [file cells-09-02557-s001.pdf]

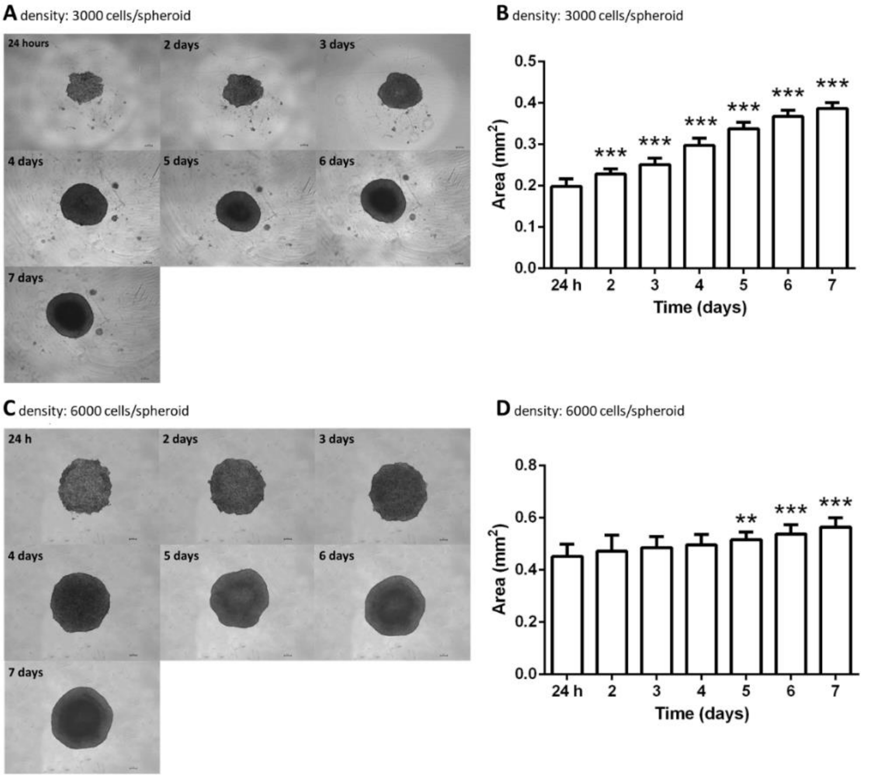

Figure S1. The growth and morphology of spheroids (planimetry) monitored during 7 days of cultivation. The surface area of spheroid size was measured every 24 h (A–B: initial density of 3000 cells/spheroid and C–D: initial density of 6000 cells/spheroid). The images were taken using an inverted microscope at 40× magnification (N = 3). Results are presented as the mean ± SD (N = 10). The statistical analysis was performed in GraphPad Prism 6, by the one-way ANOVA using the Dunnett's multiple comparisons tests, ** $p < 0.01$, *** $p < 0.001$.
